# Automated detection of myopic maculopathy using five-category models based on vision outlooker for visual recognition

**DOI:** 10.3389/fncom.2023.1169464

**Published:** 2023-04-20

**Authors:** Cheng Wan, Jiyi Fang, Xiao Hua, Lu Chen, Shaochong Zhang, Weihua Yang

**Affiliations:** ^1^College of Electronic Information Engineering, Nanjing University of Aeronautics and Astronautics, Nanjing, China; ^2^Nanjing Star-mile Technology Co., Ltd., Nanjing, China; ^3^Shenzhen Eye Hospital, Jinan University, Shenzhen, China; ^4^Shenzhen Eye Institute, Shenzhen, China

**Keywords:** myopic maculopathy, vision outlooker, visual recognition, artificial intelligence, data limitations

## Abstract

**Purpose:**

To propose a five-category model for the automatic detection of myopic macular lesions to help grassroots medical institutions conduct preliminary screening of myopic macular lesions from limited number of color fundus images.

**Methods:**

First, 1,750 fundus images of non-myopic retinal lesions and four categories of pathological myopic maculopathy were collected, graded, and labeled. Subsequently, three five-classification models based on Vision Outlooker for Visual Recognition (VOLO), EfficientNetV2, and ResNet50 for detecting myopic maculopathy were trained with data-augmented images, and the diagnostic results of the different trained models were compared and analyzed. The main evaluation metrics were sensitivity, specificity, negative predictive value (NPV), positive predictive value (PPV), area under the curve (AUC), kappa and accuracy, and receiver operating characteristic curve (ROC).

**Results:**

The diagnostic accuracy of the VOLO-D2 model was 96.60% with a kappa value of 95.60%. All indicators used for the diagnosis of myopia-free macular degeneration were 100%. The sensitivity, NPV, specificity, and PPV for diagnosis of leopard fundus were 96.43, 98.33, 100, and 100%, respectively. The sensitivity, specificity, PPV, and NPV for the diagnosis of diffuse chorioretinal atrophy were 96.88, 98.59, 93.94, and 99.29%, respectively. The sensitivity, specificity, PPV, and NPV for the diagnosis of patchy chorioretinal atrophy were 92.31, 99.26, 97.30, and 97.81%, respectively. The sensitivity, specificity, PPV, and NPV for the diagnosis of macular atrophy were 100, 98.10, 84.21, and 100%, respectively.

**Conclusion:**

The VOLO-D2 model accurately identified myopia-free macular lesions and four pathological myopia-related macular lesions with high sensitivity and specificity. It can be used in screening pathological myopic macular lesions and can help ophthalmologists and primary medical institution providers complete the initial screening diagnosis of patients.

## Introduction

Pathological myopia (PM) is one of the leading causes of visual impairment worldwide (Jonas and Panda-Jonas, 2019). The degree of myopia is usually classified as mild, moderate, or high, which often tends to develop into PM (Lu et al., 2021). Currently, myopia in children is growing rapidly worldwide, the prevalence of high myopia is also increasing, and the number of people with high myopia will increase further in the future (Morgan et al., 2012; Holden et al., 2016; Chen et al., 2021). PM has evolved from high myopia, accompanied by a series of changes, such as optic disc changes, foveal position changes, and retinopathy (Zhang et al., 2018; Meng et al., 2022). This degenerative change in the retina and choroid, known as myopic macular degeneration, is the main feature of PM. Myopic maculopathy may also lead to vision loss in patients with PM (Cho et al., 2016). Previously, the diagnosis of myopic macular degeneration relied only on the physician’s analysis of the patient’s fundus color photographs. For some underdeveloped areas, very few ophthalmologists can diagnose myopic macular degeneration (Vela et al., 2012; Resnikoff et al., 2020). The increasing number of patients with high myopia also makes this method very inefficient and urgently pushes us to find a convenient and efficient means of diagnosis.

With the rapid development of artificial intelligence (AI) technology with deep learning as the core, it is increasingly being used in the field of ophthalmology, and many researchers have used deep learning algorithms to detect common fundus diseases on fundus color images (Holden et al., 2016; Yang et al., 2019; Zhang et al., 2019, 2022; Wan et al., 2021; Zheng et al., 2021; Zhu et al., 2022). In 2019, the Singapore Eye Centre developed an AI-based deep learning system composed of a convolutional neural network pre-trained using the XGBoost algorithm to predict refractive error and myopic macular degeneration using color fundus photographs. The final area under the curve (AUC), sensitivity (SE), and specificity (SP) of macular lesion detection were 0.955, 91.9, and 87.7%, respectively (Tan et al., 2019). In 2020, a team from Japanese and Singaporean eye centers developed deep learning algorithms to identify myopic macular lesion features and automatically classify myopic macular lesions, the correct identification rates for diffuse atrophy, patchy atrophy, macular atrophy, and choroidal neovascularization were 90.18, 95.28, 97.50, and 91.14%, and an overall correct detection rate of 92.08% for PM (Du et al., 2021).

The research and development of intelligent ophthalmology has given birth to numerous automatic diagnostic systems for fundus image analysis (Gulshan et al., 2016; Burlina et al., 2017; Li et al., 2018; Zhao et al., 2022), which compensates for the shortcomings of traditional methods; achieves rapid and accurate screening of ophthalmic diseases; provides a reference for disease prevention, diagnosis, and treatment; and has significant medical value. Inspired by this, we developed an automatic macular lesion recognition system based on deep learning using the vision transformer model, which is not only efficient but also has guaranteed accuracy (ACC). This is of great importance in reducing the pressure on doctors and alleviating the shortage of medical resources.

## Materials and methods

### Ethics statements

To prevent the disclosure of invasive patient privacy, personal patient information was removed from the image collection; therefore, all color fundus images were anonymized and no relevant patient statistics were available.

### Study design and population

In our cohort study, 1,750 color fundus images from patients of different age groups and different sexes were collected from the Eye Hospital of Nanjing Medical University. Fundus images are taken by different types of non-discrete fundus cameras with the macula at the center, and the images are selected through a professional quality control process, and all images had a resolution range of 1,024 × 1,024 to 2,992 × 2,000.

### Classification and labeling of fundus images

We referenced the Meta-PM study classification system (Ohno-Matsui et al., 2015). Fundus images were classified into five categories according to myopic macular lesions: non-myopic retinal lesions, leopard fundus, diffuse chorioretinal atrophy, patchy chorioretinal atrophy, and macular atrophy, which were labeled C0, C1, C2, C3, and C4, respectively. Among them, diffuse choroidal retinal atrophy consisted of two types of diffuse choroidal atrophy around the optic papilla in peripapillary diffuse choroidal atrophy and diffuse choroidal atrophy in the macula in macular diffuse choroidal atrophy, and patchy chorioretinal atrophy consisted of patchy atrophy in the area of advanced diffuse atrophy, development of patchy atrophy due to lacunar fissures, the central concave center was rarely affected by enlargement and fusion of patchy atrophy, and visible patchy atrophy at the edge of posterior staphyloma. The four types of atrophy consisted of macular neovascularization-associated macular atrophy and C3-associated macular atrophy.

We labeled the color fundus images according to the aforementioned grading, and the labeling categories ranged from C0 to C4 in the five categories. All fundus images were authentically labeled using a double-blind method, as determined by two ophthalmologists with many years of experience in practice. When the two physicians provided the same determination for a fundus image, it was considered the final result of that image labeling. When the two doctors gave inconsistent determinations, an additional ophthalmologist made the final judgment. The fundus images obtained during the aforementioned process were collated, and the grading results obtained were used as the reference standard for this study. Among them, 310 were C0, 560 were C1, 326 were C2, 394 were C3, and 160 were C4. To exclude some subjective factors, this study used random seeds to randomly divide the dataset into independent training, testing, and validation sets. During the training process, the training and validation datasets were used for model tuning, and the test dataset was used to evaluate the effects of the trained model. The datasets were divided at a ratio of 8:1:1, and the final data were divided (Table 1).

**TABLE 1:** Dataset division of five categories from C0 to C4.

Category	Training dataset	Test dataset	Validation dataset
C0	248	31	31
C1	448	56	56
C2	262	32	32
C3	316	39	39
C4	128	16	16

After the fundus images were grouped, a series of data enhancement processes was performed on the training dataset to prevent overfitting during the training process. First, the images were normalized; panning, scaling, and rotation were performed; the hue, saturation, and parametric brightness and contrast of the input images were randomly changed; and the images were scaled down to a resolution of 256 × 256 according to the optimal input size for the model. The validation dataset was also subjected to simple image cropping and normalization. Data enhancement can effectively improve the adaptability of the network to images of the same class, but with some differences, such as the generalization ability of the network. Then, the image data were packaged and fed into the network model.

### Vision outlooker for visual recognition model

Recently, deep learning technology has developed rapidly. Since the early AlexNet (Vintch et al., 2012), the network has emerged in the competition. Subsequently, a series of excellent convolutional neural network models has been developed (Simonyan and Zisserman, 2014; Zeiler and Fergus, 2014; Iandola et al., 2016; Larsson et al., 2016; Bo et al., 2017; Szegedy et al., 2017). To date, there has been an increase in the number of transformer models (Dosovitskiy et al., 2020; Liu et al., 2021). For the development of a vision transformer (Vaswani et al., 2017), we have conducted a lot of research and selected the Vision Outlooker for Visual Recognition (VOLO) (Yuan et al., 2022) model–a powerful model architecture for visual recognition with better results so far–for experimentation, which proposes a new lightweight attention mechanism, the Outlooker, which can efficiently encode fine-level information and is designed with a two-phase architecture that considers more fine-grained encoding of token representations and global information aggregation. We used the Pytorch (Paszke et al., 2019) framework for model building and selected the EfficientNetV2 (Tan and Le, 2021) and ResNet50 (He et al., 2016) models to compare the results with those of the VOLO-D2 model. All models were trained with hyperparameters using weights pretrained from the ImageNet dataset (Deng et al., 2009), and used a unified approach to data enhancement.

**FIGURE 1:**
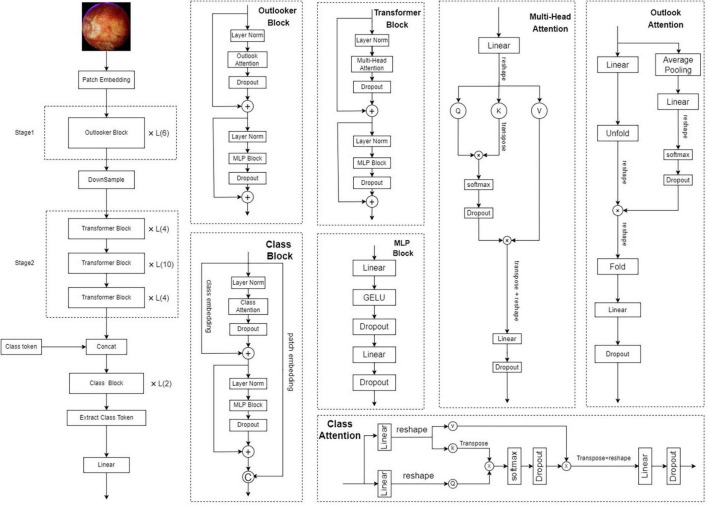
Architecture of the VOLO networks. VOLO, vision outlooker for visual recognition.

**FIGURE 2:**
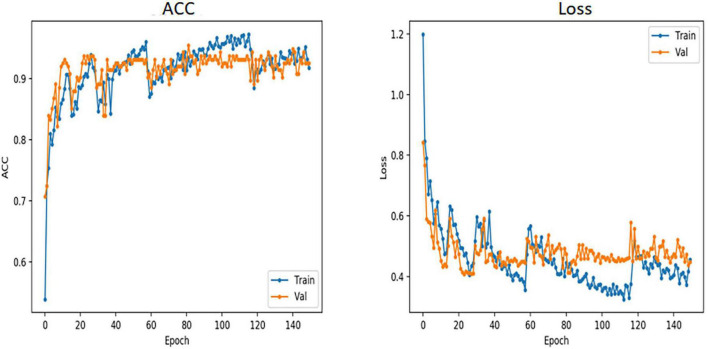
Accuracy and loss curves of VOLO-D2. VOLO, vision outlooker for visual recognition; ACC, accuracy; Train, training; Val, validation; Epoch, train once with all the samples in the training dataset.

During training, a smaller training dataset increases the probability of overfitting problems. To prevent overfitting and enhance the robustness of the model, we enhanced the training data in several different ways, including random horizontal and vertical flipping; random orientation rotation; and modification of brightness, contrast, and saturation to cause color interference. In each iteration, 80% of the total samples were used for training and 10% for validation. The total number of iterations in the training process was 100, and we used the AdamW optimizer (Loshchilov and Hutter, 2017) with a batch size of 32 and weight decay of 0.05 (Jiang et al., 2021). The initial learning rate was set to 0.0001, and the learning rate was decayed using a cosine annealing decay strategy. To quickly bring the ACC to a reasonable range, the first 10 iterations were linearly ascending (Touvron et al., 2021a). The model structure and learning curve of VOLO-D2 are shown in Figures 1, 2.

Experimental hardware: The central processing unit used was a 2.80-GHz Intel^®^ Xeon^®^ E5-1603 version 4; the graphics processing unit was the NVIDIA GeForce GTX1080 with 8-GB RAM. Experimental software: The model was constructed using PyTorch and Python.

### Analysis of the effects of the model

In our study, which has a multicategorical problem, we used two methods to evaluate the effects of the model. One of the methods was to evaluate the overall effect using ACC with the kappa coefficient as an evaluation metric. The kappa coefficient was calculated based on the confusion matrix, which is generally between 0 and 1. The higher its value, the better the ACC of the model classification. The kappa coefficient was calculated as follows:


(1) $$\text{k}=\frac{p_{0}-p_{e}}{1-p_{e}}$$

(2) $$p_{e}=\frac{a_{1}\times b_{1}+a_{1}\times b_{1}+\ldots +a_{c}\times b_{c}}{n\times n}$$

where *p*_0_ represents the total classification ACC, *a*_*i*_ is the number of true samples of class i, and *b*_*i*_ is the number of predicted samples of class i.

Another approach is to convert the multi-classification problem into multiple independent binary classification problems, i.e., to identify myopia-free retinal lesions, class C0 is marked as a positive sample, and the remaining classes (C1, C2, C3, and C4) of lesions are negative samples. For the evaluation index of the dichotomous problem, the numbers of true positive (TP), true negative (TN), false positive (FP), and false positive (FN) samples were first determined using a confusion matrix, and then the ACC, SE, SP, positive predictive value (PPV), and negative predictive value (NPV) were calculated. The specific calculation of each evaluation index is as follows:


(3) $$\text{ACC}=\frac{T\,P+T\,N}{T\,P+F\,N+T\,N+F\,P}$$

(4) $$\text{SE}=\frac{T\,P}{T\,P+F\,N}$$

(5) $$\text{SP}=\frac{T\,N}{T\,N+F\,P}$$

(6) $$\text{PPV}=\frac{T\,P}{T\,P+F\,P}$$

(7) $$\text{NPV}=\frac{T\,N}{T\,N+F\,N}$$

To enable a detailed understanding of the performance of the model for each type, we used receiver operating characteristic (ROC) curves to evaluate the classification performance. Additionally, the AUC values calculated using the ROC curve were used to evaluate the performance of the classifier. The closer the AUC is to 1.0, the better the classification performance.

## Results

**FIGURE 3:**
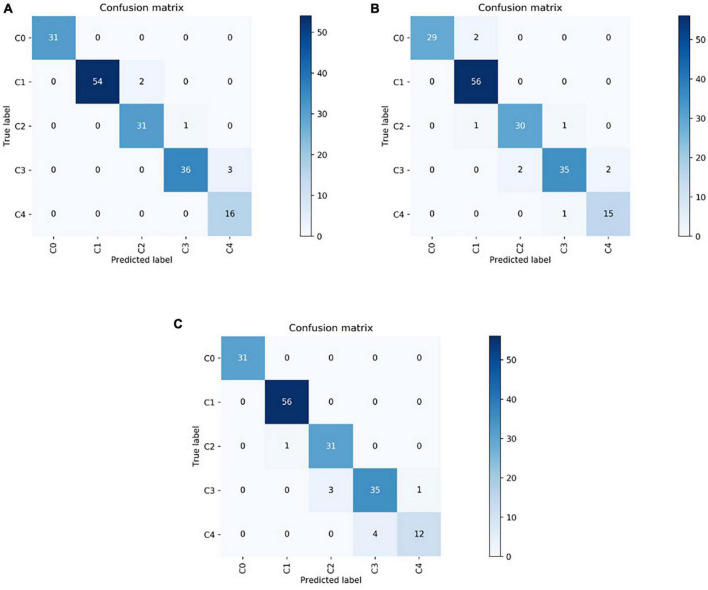
The test dataset results are shown as a confusion matrix in which the vertical axis represents the true labels and the horizontal axis represents the predicted labels. **(A)** Confusion matrix of VOLO-D2. **(B)** Confusion matrix of EfficientNetV2-S. **(C)** Confusion matrix of ResNet50. VOLO, vision outlooker for visual recognition.

In this study, 174 fundus images (31 images of class C0, 56 images of class C1, 32 images of class C2, 39 images of class C3, and 16 images of class C4) were randomly selected for external testing. The VOLO-D2 model achieved an overall recognition rate of 96.60% and kappa coefficient of 95.60% in the test set, both of which proved that the model was significantly effective in identifying pathologically related macular lesions. The VOLO-D2 five-category model for 31 images of class C0 and 16 images of class C4 showed correct results, with the remaining numbers of correctly diagnosed images of C1, C2, and C3 being 54, 31, and 36, respectively. For the EfficientNetV2-S five-category model for 56 images of class C1, the results were correct; the numbers of correct diagnostic images for C0, C2, C3, and C4 were 29, 30, 35, and 15, respectively. In the ResNet50 five-category model for 31 images of class C0 and 56 images of class C1, the results were correct, and the numbers of correct diagnosis images for C2, C3, and C4 were 31, 35, and 12, respectively. The confusion matrices of the VOLO-D2, EfficientNetV2-S, and ResNet50 models are shown in Figures 3A–C.

The aim of this study was to correctly diagnose non-myopic retinal lesions and the four PM-related macular degeneration, for which we show a comparison of the performance of the VOLO-D2 model and the other two models in recognizing each type of image. The evaluation results are shown in Table 2. For the diagnosis of images without myopic retinal degenerative lesions (C0), the VOLO-D2 model had a very good performance of 100% in all metrics.

**TABLE 2:** Evaluation of the index results of the three models.

Model	Evaluation indicators	C0	C1	C2	C3	C4
VOLO-D2	Sensitivity	100%	96.43%	96.88%	92.31%	100%
	Specificity	100%	100%	98.59%	99.26%	98.10 %
	PPV	100%	100%	93.94%	97.30%	84.21%
	NPV	100%	98.33%	99.29%	97.81%	100%
	AUC	100%	98.21%	97.73%	95.78%	99.05%
	Kappa			95.60%		
	Accuracy			96.60%		
EfficientNetV2-S	Sensitivity	93.54%	100%	93.75%	89.74%	93.75%
	Specificity	100%	97.46%	98.59%	98.52%	98.73%
	PPV	100%	94.92%	93.75%	94.59%	88.24%
	NPV	98.62%	100%	98.59%	97.08%	99.36%
	AUC	96.77%	98.73%	96.17%	94.13%	96.24%
	Kappa			93.30%		
	Accuracy			94.80%		
ResNet50	Sensitivity	100%	100%	96.88%	89.74%	75%
	Specificity	100%	99.15%	97.89%	97.04%	99.37%
	PPV	100%	98.25%	91.18%	89.74%	92.31%
	NPV	100%	100%	99.29%	97.04%	97.52%
	AUC	100%	99.58%	97.38%	93.39%	87.18%
	Kappa			93.30%		
	Accuracy			94.80%		

PPV, positive predictive value; NPV, negative predictive value; AUC, area under the curve; VOLO, vision outlooker for visual recognition.

For the diagnosis of leopard fundus images (C1), the model had an AUC of 98.21%, an SE of 96.43%, and an NPV of 98.33%; both SP and PPV were 100%. For the diagnosis of diffuse chorioretinal atrophy images (C2), the model had an AUC of 97.73, SE of 96.88, SP of 98.59, PPV of 93.94, and NPV of 99.29%. For the diagnosis of patchy chorioretinal atrophy images (C3), the model had an AUC of 95.78, SE of 92.31, SP of 99.26, PPV of 97.30, and NPV of 97.81%. For the diagnosis of macular atrophy images (C4), the model had an AUC of 99.05, PPV of 84.21, and NPV of 98.10%; it performed very well in terms of SP and SE, both of which were 100%. These data illustrate the high consistency of the VOLO-D2 model. The SE of the EfficientNetV2-S model for diagnosing all categories was >89%, with a slightly weaker overall performance and higher consistency. Although the ResNet50 model had high SE and SP in the diagnosis of non-myopic retinal degenerative lesions and leopard-eye fundus lesions, the SE was only 75% in the diagnosis of macular atrophy lesions, indicating poor performance.

**FIGURE 4:**
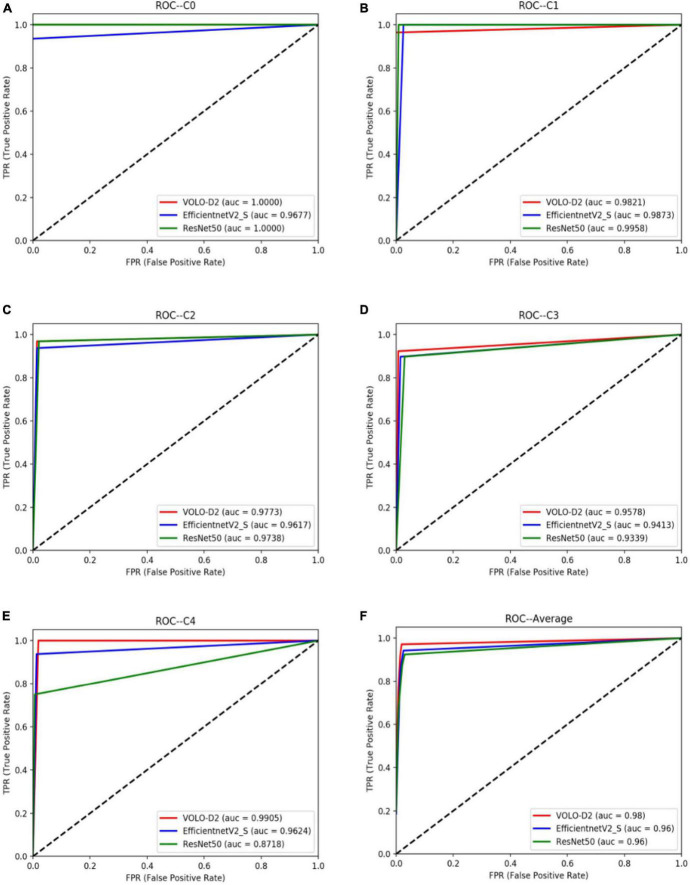
ROC curves of the VOLO-D2, EfficientNetV2-S, and ResNet50 models for myopia-free macular lesions and four pathological myopia-related macular lesions. **(A)** ROC curves and AUC value of C0. **(B)** ROC curves and AUC value of C1. **(C)** ROC curves and AUC value of C2. **(D)** ROC curves and AUC value of C3. **(E)** ROC curves and AUC value of C4. **(F)** ROC curves and AUC value of the average of all categories. ROC, receiver operating characteristic; VOLO, vision outlooker for visual recognition.

As shown in Table 2, the VOLO-D2 model was better than the EfficientNetV2-S and ResNet50 models in terms of SE and SP for C0, C2, C3, and C4. The ROC curve between each model (Figure 4) also shows that the VOLO-D2 model was excellent in diagnosing non-myopic retinal degenerative lesions, diffuse choroidal retinal atrophy, patchy choroidal retinal atrophy, and macular atrophy.

**FIGURE 5:**
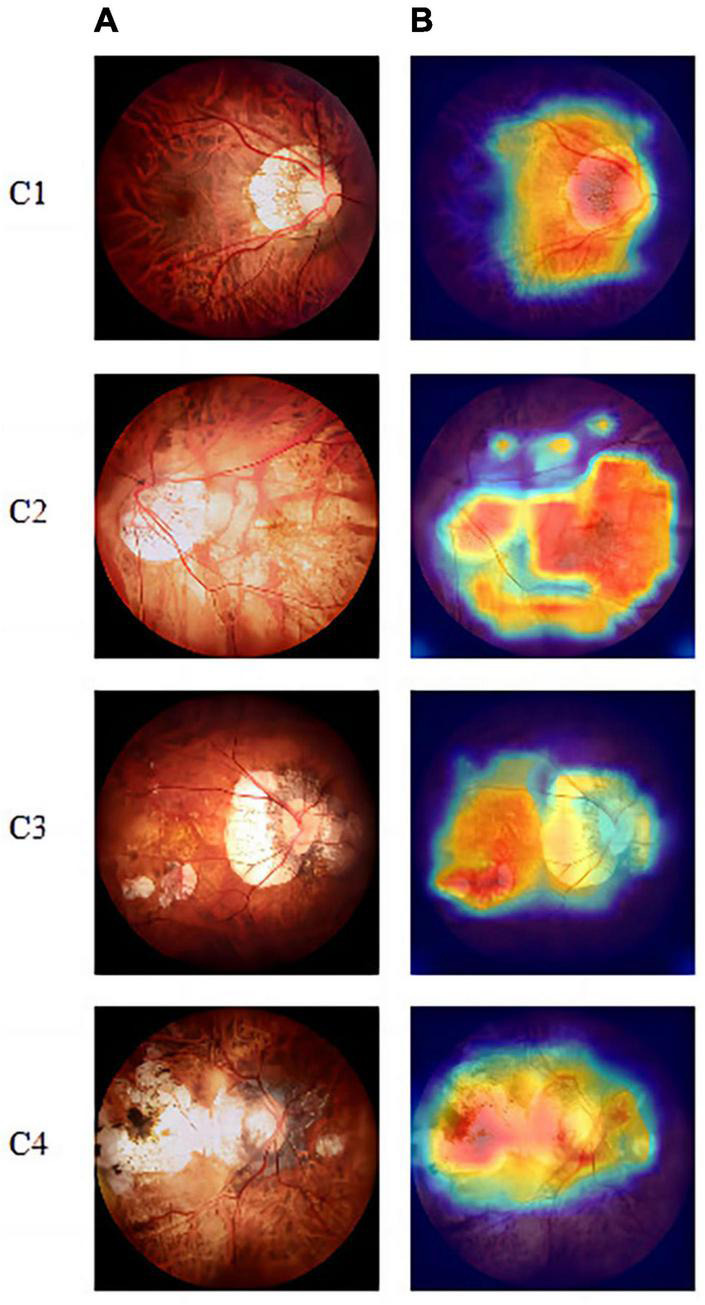
Visualization-based diagnosis by the VOLO-D2 model. **(A)** Original image. **(B)** Grad-CAM. Grad-CAM shows that the model focused on the myopic maculopathy area. From left to right, we can see the original fundus images of the four lesions along with the corresponding gradient activation heat maps. Grad-CAM, gradient-weighted class activation mapping.

We also used gradient-weighted class activation mapping to analyze the lesion areas in the fundus images, as shown in Figures 5A, B. The warmer the colors in the heat map, the greater is the impact on the classification prediction results.

## Discussion

Myopia has become the most common public health problem worldwide, and myopic macular lesions are common complications caused by myopia. With the development of myopia, macular lesions further trigger vision decline. Therefore, it is important to detect PM at an early stage, as this has important clinical significance. At present, because of the large number of patients with myopia and the lack of ophthalmic myopia experts, the initial diagnosis of this pathological myopic macular lesion is extremely important.

In 2020, the Vision Transformer (ViT) was proposed, which pioneered the application of Transformer in the computer vision (CV) field, which have demonstrated its effectiveness on multiple CV tasks. The visual Transformers have strong feature extraction ability and achieve outstanding performance in multiple tests compared with Convolution Neural Networks (CNNs). Based on this research, a large number of excellent models of the visual Transformers such as TNT (Salvatori et al., 2021), PVT (Wang et al., 2021), CaiT (Touvron et al., 2021b), Swin Transformer and VOLO have been proposed. In this study, the VOLO model was selected to classify non-myopic retinal lesions and four categories of pathological myopic maculopathy. Compared with other the visual Transformers models, VOLO exhibits better feature extraction ability and can improve the accuracy of classification recognition. In this study, we compare two excellent CNNs models in order to demonstrate that the visual Transformers model is very effective in medical fundus image diagnosis.

Although machine learning has shown great potential, a number of significant data-related problems plague the application of machine learning in helping diagnosis and prognosis of myopic maculopathy. Our research method involved collecting the eyes of limited number of patients with myopia and data training with various deep learning models. Our findings prove that the VOLO-D2 model has high diagnostic ACC and SE. Furthermore, the assessment of the test set proved that our PM image classification system has good generalization capabilities. Using our system, the patient’s symptoms can be preliminarily determined from the perspective of medical ophthalmology, so doctors can further diagnose and treat the disease and prevent further development of blindness.

Some researchers had also done research on multiple macular disorders. Li et al. (2022) used DCNN-DS model to detect no myopic maculopathy, tessellated fundus, and pathologic myopia, and the validation accuracies on the two external testing datasets were 96.3 and 93.0%, respectively. Tang et al. (2022) used ResNet-50 model to develop the META-PM study categorizing system, and the mean accuracy was 0.9119 ± 0.0093 on the five categories. The overall accuracy of the VOLO-D2 model in this study is 96.60% on the five categories, but the number of images in the external test set is small, only 176. In the experiments, the results were also compared with two high-quality CNNs models.

Figure 3 and Table 1 show that the VOLO-D2 model performs better in the overall recognition accuracy and kappa value of the test set compared with the EfficientNetV2-S and ResNet50 models. It can be demonstrated that the VOLO-D2 model has a deeper feature understanding of the complexity of macular lesion images than the CNNs model. These deeper features can improve the accuracy of the model in recognizing macular lesion images. Figure 4 shows visually that the VOLO-D2 model performs somewhat worse in identifying leopard’s fundus compared to the EfficientNetV2-S and ResNet50 models, misdiagnosis as other pathologies occurred.

According to the hot diagram of macular lesions shown in Figure 5, the VOLO-D2 model’s interest in each lesion has laid the foundation for us to further study the cutting and diagnosis of the macular lesion area. As shown in the confusion matrix in Figure 3, there were several cases of identification errors in the VOLO-D2 model, especially when the diagnosis of patchy chorioretinal atrophy, and errors were also diagnosed as macular atrophy. It is difficult to distinguish between the two when the colors are similar to the sluggish areas. However, this is satisfactory for the overall recognition rate. Therefore, our model can be used for preliminary disease screening in cooperation with ophthalmologists.

Our study has certain limitations. First, owing to the difficulty of data collection, the amount of data in this experiment is small, and we cannot assess its generalization. Second, there is further room for improvement in the incorrect diagnosis of some lesions. Next, we will continuously improve the model to improve ACC, use more new datasets for training and testing, and further use image segmentation methods to determine the pathogen area to improve auxiliary ophthalmologists for diagnosis.

## Conclusion

Overall, the five-category model of VOLO-D2 has high SE and SP for the diagnosis of non-myopic retinal lesions and four PM-associated maculopathies. We hope that it can help identify and treat patients early and protect them from low vision and blind diseases caused by myopic macular lesions. Additionally, we hope to help ophthalmologists reduce part of their work.

## Data availability statement

The raw data supporting the conclusions of this article will be made available by the authors, without undue reservation.

## Ethics statement

Ethical review and approval was not required for the study on human participants in accordance with the local legislation and institutional requirements. Written informed consent from the patients/participants or patients/participants’ legal guardian/next of kin was not required to participate in this study in accordance with the national legislation and the institutional requirements.

## Author contributions

CW and JF analyzed and discussed the data and drafted the manuscript. XH analyzed and discussed the data and trained the model. LC collected and labeled the data. SZ and WY designed the research, collected and labeled the data, and revised the manuscript. All authors contributed to the article and approved the submitted version.
